# Multi-joint and multi-planar lower-limb moment prediction from four inertial measurement units with target-subject adaptation

**DOI:** 10.3389/fbioe.2026.1938657

**Published:** 2026-10-21

**Authors:** Xinyu Fan, Tao Ma, Ningbo Yu, Jianda Han, Jiayin Guan, Monan Ni, Tao Sun

**Affiliations:** 1 Key Laboratory of Mechanism Theory and Equipment Design of Ministry of Education and Institute for Embodied Intelligence, Tianjin University, Tianjin, China; 2 College of Artificial Intelligence, Nankai University, Tianjin, China; 3 Tianjin Medical Devices Quality Supervision and Testing Center, Tianjin, China

**Keywords:** deep learning, domain adaptation, joint moment prediction, multi-task learning, wearable sensors

## Abstract

**Introduction:**

Accurate and continuous prediction of multi-planar lower-limb joint moments is crucial for clinical rehabilitation, sports science, and assistive device design. However, gold-standard methods rely on bulky, expensive laboratory-bound equipment, and existing wearable solutions are often limited to single-joint or single-plane predictions, or require dense sensor arrays impractical for daily living.

**Methods:**

To address these limitations, we propose an end‐to‐end multi-task temporal convolutional network framework. Using only four inertial measurement units sparsely placed on the pelvis and lower limb, the framework simultaneously predicts five continuous joint moments: hip and knee moments in the coronal plane, and hip, knee, and ankle moments in the sagittal plane. A hard‐parameter-sharing temporal convolutional backbone extracts cross‐joint temporal features, while point‐wise channel attention enables task‐specific feature reweighting. Adaptive batch normalization (AdaBN) further enables target-subject adaptation using a 10‐s segment of unlabeled level‐walking data. We evaluated the model on 14 participants performing level walking, stair ascent, and stair descent under nested subject‐level leave‐one‐subject‐out cross‐validation.

**Results:**

The proposed model achieved the highest numerical overall R^2^ among models evaluated without target‐subject adaptation and significantly outperformed four of the six external baselines; AdaBN further improved the overall R^2^ to 0.759 ± 0.032. Ablation experiments showed that hard parameter sharing, point‐wise channel attention, and AdaBN increased the overall R^2^ by ΔR^2^ = 0.017, 0.026, and 0.032, respectively (all Holm‐adjusted p < 0.05). Supplementary evaluation on an independent public dataset yielded R^2^ values of 0.704–0.840 across four joint‐moment targets.

**Discussion:**

These findings demonstrate the feasibility of continuous multi‐joint and multi‐planar moment prediction using a sparse wearable sensor configuration under controlled laboratory conditions and support further evaluation in larger and clinically diverse cohorts and in out‐of‐laboratory settings.

## 1 Introduction

Lower-limb joint moments quantify the mechanical output of the musculoskeletal system against external loads, serving as a critical metric for evaluating the forces exerted on internal joint soft tissues during human movement (Holder et al., 2020). In clinical and rehabilitation medicine, joint moments provide a vital basis for assessing disease progression and monitoring the rehabilitation of chronic musculoskeletal disorders, such as knee osteoarthritis (Miyazaki et al., 2002; Bennell et al., 2011; Yang et al., 2024; Ma et al., 2023; 2025). In sports science, quantifying these moments assists in movement optimization and the prevention of sports injuries (Carter et al., 2026). Furthermore, in the design of assistive rehabilitation devices, joint moment data can be utilized to optimize control strategies and facilitate human-machine synergy (Nuckols et al., 2020).

Currently, the gold standard for measuring lower-limb joint moments involves collecting kinematic and kinetic data via optical motion capture systems and force plates (Lencioni et al., 2019), followed by computations using inverse dynamics algorithms or musculoskeletal models (Zhang et al., 2024; Uhlrich et al., 2023). While this approach is widely adopted in biomechanics, its reliance on expensive equipment, cumbersome system calibration procedures, and computationally complex musculoskeletal modeling largely confines it to controlled laboratory environments (Muro-de-la-Herran et al., 2014). Moreover, participants tend to subconsciously alter their gait in laboratory settings, resulting in a lack of sufficient ecological validity for most lab-based assessments. Data acquired during these brief, idealized sessions struggle to accurately reflect the natural gait characteristics of participants under daily, prolonged mechanical loading (Renggli et al., 2020; Takayanagi et al., 2019). Consequently, researchers have increasingly focused on exploring highly portable and cost-effective alternatives for joint moment acquisition.

Advances in sensing and machine learning now make it feasible to acquire joint moments in uncontrolled, natural environments. Prior work has used wearable devices, including inertial measurement units (IMUs) (Tang et al., 2025), encoders, pressure insoles (Molinaro et al., 2024b), and surface electromyography sensors (Zhang et al., 2022), to estimate lower-limb joint moments, yet most efforts have targeted a single joint (Molinaro et al., 2022; 2024a). For instance, hip sagittal moments have been predicted from encoders and IMUs for exoskeleton control (Molinaro et al., 2024a), and knee adduction moments have been estimated for quantitative assessment of osteoarthritis (Stetter et al., 2020; Feng et al., 2025). However, human gait emerges from coordinated multi-joint action (Pan et al., 2025); analyzing a single joint moment misses whole-limb compensatory patterns and can bias screening and assessment. Consequently, some studies have attempted to predict hip, knee, and ankle sagittal moments simultaneously to capture multi-joint synergy (Carter et al., 2026; Tang et al., 2025; Hossain et al., 2022). Although these efforts broaden joint coverage, they remain limited to the sagittal plane and do not meet the clinical need for multi-planar kinetic information. In knee osteoarthritis, for example, patients redistribute loads across the sagittal and coronal planes of the knee (Creaby, 2015) and between joints (Pan et al., 2025; Hall et al., 2017) to relieve pain, while the coronal hip moment becomes abnormal (Ma et al., 2025) and ankle propulsion changes (Pan et al., 2025; Ro et al., 2019). Continuous monitoring of key sagittal- and coronal-plane moments across the hip, knee, and ankle may therefore provide valuable biomechanical information for future screening and rehabilitation assessment.

Motivated by the need for multi-joint and multi-planar kinetic information, previous studies have further extended wearable joint-moment estimation to simultaneous prediction across multiple joints and anatomical planes. Lee and Park (2020) and Mundt et al. (2020) investigated multi-joint and multi-planar moment prediction during level walking, although both approaches relied on segmented and time-normalized gait data and used feedforward neural networks without explicit temporal modeling. Altai et al. (2023) further extended this task to multiple locomotion modes using four IMUs to predict five lower-limb joint moments; however, the target set did not include the knee adduction moment (KAM), an important biomechanical indicator of medial knee joint loading in knee osteoarthritis, and prediction was likewise based on stride-segmented, time-normalized full-cycle waveforms. Collectively, these studies still rely on gait segmentation and time-normalized inputs, limiting their applicability to real-time continuous prediction, while the coverage of clinically relevant multi-planar joint moments remains incomplete.

Beyond these task-level limitations, cross-subject generalization presents a further challenge for wearable joint-moment prediction. Altai et al. (2023) reported reduced prediction performance under leave-subject-out evaluation, while Moghadam et al. (2023) similarly observed lower prediction accuracy in inter-subject than intra-subject evaluations. Some approaches have combined multi-source inputs, such as surface electromyography and joint-angle information, with supervised transfer learning to improve cross-subject generalization (Zhang et al., 2022). However, such configurations increase sensor complexity, while supervised transfer learning requires reference joint-moment labels from target participants for adaptation. These limitations motivate adaptation strategies that can account for inter-subject variability without requiring labeled joint-moment data from new subjects.

To address these task-level and cross-subject challenges, this study develops a multi-task wearable framework based on a sparse four-IMU configuration for continuous prediction of five lower-limb joint moments during level walking, stair ascent, and stair descent, including sagittal- and coronal-plane moments at the hip and knee and the sagittal-plane moment at the ankle. These targets cover key joint-moment components relevant to lower-limb musculoskeletal assessment. The principal methodological contribution of this study is a unified task-oriented framework that combines shared temporal learning across multiple joint-moment targets, task-specific feature reweighting for individual moment predictions, and label-free adaptation to new subjects. Specifically, a hard-shared causal TCN learns common temporal features across prediction targets, task-specific point-wise channel attention reweights these features for individual joint moments, and an AdaBN-based adaptation procedure recalibrates feature statistics using only a 10-s segment of unlabeled target-subject walking data.

## 2 Materials and methods

### 2.1 Participant information

This study recruited 14 healthy participants (9 males and five females; age 24.8 ± 3.0 years; height 172.7 ± 10.6 cm; mass 66.3 ± 8.5 kg). None had a history of neurological or musculoskeletal disorders, and all reported no lower limb injuries within the 6 months prior to the experiment. The experimental protocol was approved by the ethics committee of Nankai University (Approval No. NKUIRB2026081). All participants fully understood the experimental procedures and potential risks, and they provided written informed consent before participation.

### 2.2 Sensor system configuration

As illustrated in Figure 1, a wearable sensor system tailored for activities of daily living was developed for this study. The system comprises customized nine-axis IMUs (WitMotion, Shenzhen, China) and a host application developed using the Qt framework. The IMU enclosures feature an ergonomic design and are secured with breathable, elastic straps to minimize interference with natural gait. Furthermore, the enclosure design constrains the attachment orientation of the IMUs on the limb segments, thereby reducing rotational variability during sensor placement. The host software runs on a personal computer or mobile phone and collects sensor data via Bluetooth at a sampling frequency of 100 Hz. Only tri-axial acceleration and angular velocity were used as model inputs; magnetometer signals were excluded to avoid environmental magnetic interference, resulting in six channels per IMU and 24 channels in total.

**FIGURE 1:**
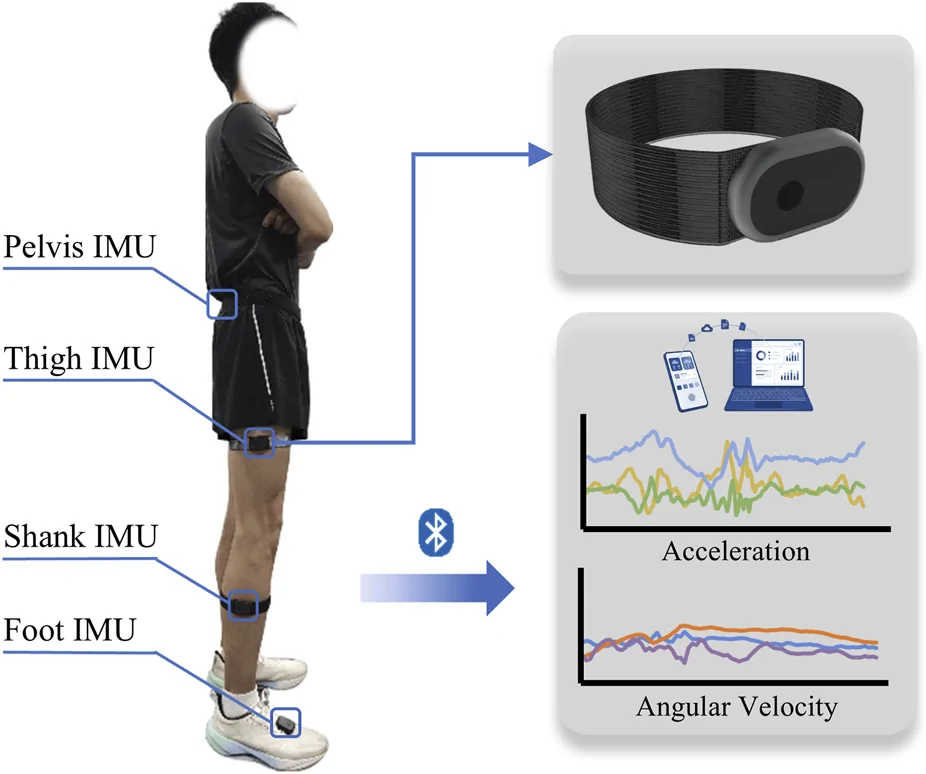
Overview of the wearable sensing system and sensor placement on the lower limbs.

Four IMUs were attached to the pelvis (midpoint between the left and right posterior superior iliac spines), right thigh (midpoint between the greater trochanter and the lateral femoral epicondyle), right shank (midpoint between the fibular head and the tip of the lateral malleolus), and the dorsum of the right foot (second metatarsal) of the participants. The rationale for this configuration relies on modeling the human lower extremity as a serial multi-rigid-body kinematic chain comprising the pelvis, thigh, shank, and foot. Acquiring the continuous posture changes of these four core rigid segments in the global space provides coverage of the major lower-limb segments (Klein et al., 2026; Prisco et al., 2024).

### 2.3 Experimental protocol

Data collection was conducted in a standard biomechanics laboratory to obtain the reference joint moments required for supervised learning. The laboratory was equipped with an optical motion-capture system comprising 10 infrared cameras (Nokov, Beijing, China) and four embedded three-dimensional force plates (Bertec, Ohio, United States of America). The motion-capture system and force plates were both sampled at 100 Hz and were hardware-synchronized to ensure temporal alignment. The IMUs were also sampled at 100 Hz, and the IMU data were temporally aligned with the motion-capture and force-plate data using timestamps recorded during data acquisition. Although the 100-Hz sampling frequency was lower than that commonly used in some inverse-dynamics studies, it provided sufficient temporal resolution for the self-selected level-walking and stair-locomotion tasks examined in this study.

Before the experiment, reflective markers were attached to the relevant anatomical landmarks of each participant based on the OpenSim gait2392 Marker Set. After brief instructions on sensor placement, participants independently donned the IMUs at the designated locations to approximate a self-donning procedure. After donning the sensors, participants stood still in the prescribed orientation for 3 s for static model calibration.

Before formal data collection, all participants completed sufficient familiarization trials. The experimental protocol comprised stair-ascent and stair-descent trials, from which three locomotion modes were evaluated: stair ascent, stair descent, and level walking. All locomotion was performed at a self-selected natural speed. The experimental staircase consisted of four steps, each with a step height of 18 cm and a tread depth of 30 cm, and was not equipped with handrails. For stair ascent and descent, the force plates were embedded flush with the surfaces of the corresponding steps. Before formal recording, the starting position was adjusted according to each participant’s natural stepping pattern so that the stair task could be performed naturally without deliberately targeting the force plates. Within the instrumented stair section, two successive right-foot initial contacts on separate force plates were used to delimit one complete stair-locomotion gait cycle, with each right-foot stance phase fully captured by its corresponding force plate for inverse dynamics analysis. Stair-descent trials additionally included subsequent level walking, from which one complete right-limb level-walking gait cycle was extracted, with its initial contact occurring approximately 0.8 m beyond the final stair after the immediate stair-to-level transition steps had been excluded. Each stair task was repeated until six valid trials satisfying the predefined quality-control criteria were obtained, with adequate rest provided between trials to minimize fatigue-related effects. Across the recorded trials, the mean achieved self-selected locomotion speeds were 0.515 ± 0.068 m/s for level walking, 0.459 ± 0.034 m/s for stair ascent, and 0.441 ± 0.043 m/s for stair descent. The overall experimental data-collection and processing workflow is shown in Figure 2.

**FIGURE 2:**
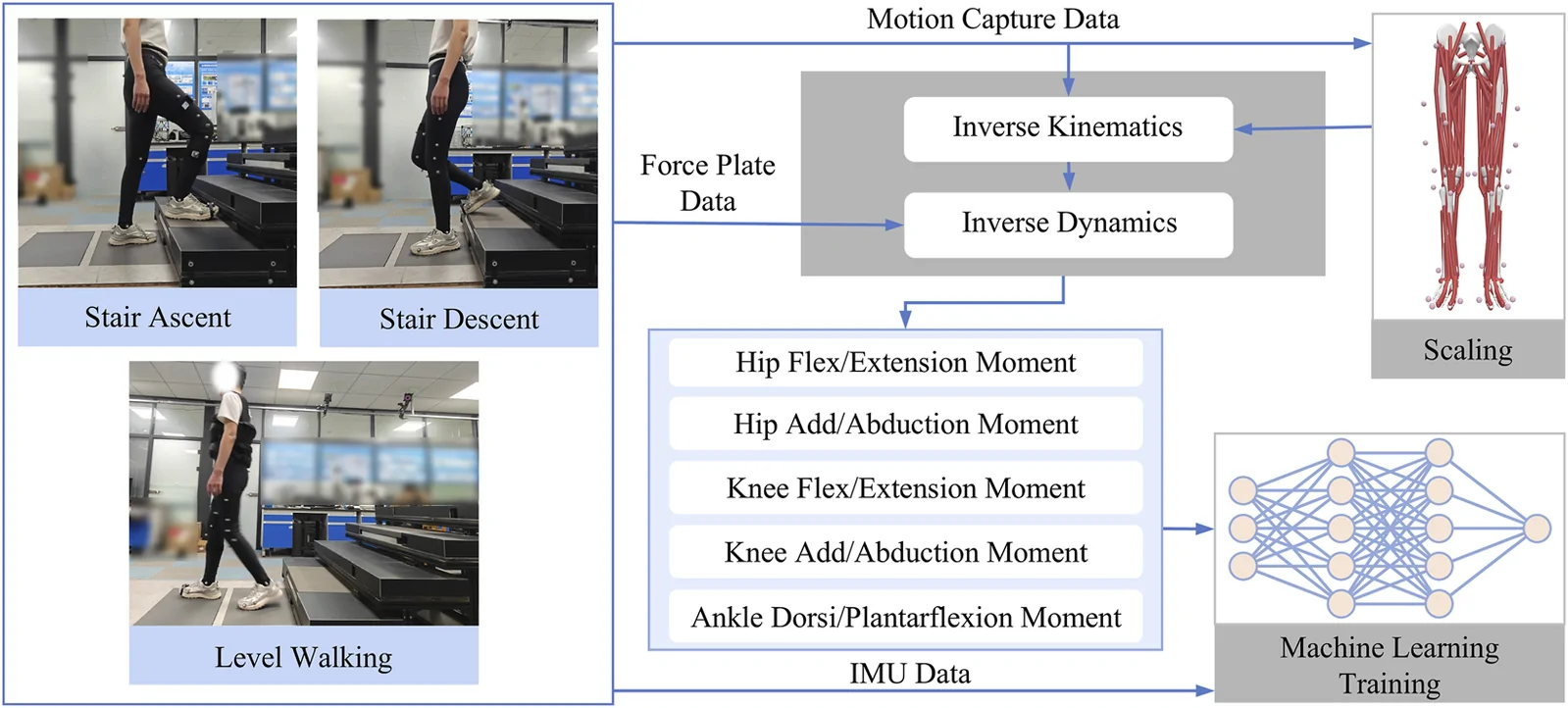
Schematic overview of the integrated framework combining experimental data collection, biomechanical analysis, and machine learning.

### 2.4 Data processing pipeline

Reference joint moments used as supervised learning labels were calculated using OpenSim 4.5 (SimTK, Stanford, CA, United States of America). Short gaps in marker trajectories caused by line-of-sight occlusion were reconstructed using spline interpolation in XINGYING software (Nokov, Beijing, China). Interpolation was applied only when the duration of a continuous gap did not exceed 10% of the corresponding gait cycle (Lencioni et al., 2019). The marker trajectories and force-plate data were subsequently low-pass filtered using a fourth-order Butterworth filter with a cutoff frequency of 15 Hz. No additional filtering, smoothing, or resampling was applied to the IMU acceleration and angular-velocity signals before model input.

Joint moments were calculated using the full-body musculoskeletal model developed by Rajagopal et al. (2016). In the original model, knee flexion/extension is represented by an independent generalized coordinate. Following previous OpenSim-based approaches, an additional independent rotational degree of freedom for knee adduction/abduction was introduced to enable calculation of the coronal-plane knee moment (Cui et al., 2019; Goell et al., 2025). The generic model was scaled to each participant using the OpenSim Scale Tool based on the static calibration trial and anthropometric information. Scaling quality was assessed using the root mean square (RMS) error between model and experimental marker positions, with Scale RMS marker errors below 4 mm for all participants.

Continuous joint kinematics were subsequently obtained using the OpenSim Inverse Kinematics Tool by minimizing the weighted positional errors between experimental and model markers. The quality of the inverse-kinematics solutions was assessed using the RMS and maximum marker errors, which remained below the recommended OpenSim guidelines of 20 mm and 40 mm, respectively, for all included trials. For inverse dynamics, the kinematic data obtained from inverse kinematics and the external loads measured by the force plates were provided to the OpenSim Inverse Dynamics Tool to calculate the net internal joint moments of the hip, knee, and ankle according to the OpenSim model coordinate conventions. As an additional biomechanical plausibility check, the coronal-plane knee moment profiles during level walking were compared with previously reported knee adduction moment patterns in healthy adults, accounting for differences in internal/external moment conventions, and showed comparable stance-phase waveform characteristics and magnitudes (Ota et al., 2017; Zhang et al., 2022).

Gait events were identified from the vertical ground reaction force (vGRF). Initial contact (IC) was defined as the first time point at which the vGRF exceeded 20 N, whereas toe-off (TO) was defined as the time point during late stance at which the vGRF decreased below 20 N. A complete gait cycle was defined from initial contact of the right foot to the subsequent initial contact of the same foot.

Five right-limb joint moments were selected as prediction targets: hip flexion/extension, hip adduction/abduction, knee flexion/extension, knee adduction/abduction, and ankle dorsiflexion/plantarflexion moments (D'Souza et al., 2022). Moment signs followed the coordinate conventions of the OpenSim model: hip and knee flexion, hip and knee adduction, and ankle dorsiflexion were defined as positive, whereas the corresponding opposite directions were negative. All joint moments were normalized to body mass and expressed in N·m/kg (Moisio et al., 2003).

Trials were excluded if continuous marker gaps exceeded 10% of the corresponding gait cycle, the right foot did not land completely within the active area of the force plate, packet loss or other data-integrity problems were identified in the IMU recordings, or reliable temporal alignment among the motion-capture, force-plate, and IMU data could not be achieved. A total of four trials were excluded because of IMU data-integrity or synchronization problems.

### 2.5 Dataset construction

To prepare the processed IMU features and reference joint moments for subsequent deep learning modeling, a sliding-window approach was used to construct model samples. The continuous time series of each trial was segmented independently using a window length of 105 samples, corresponding to 1.05 s, with a stride of five samples. The IMU data within each window constituted an input tensor $X\in R^{24\times 105}$ , while the corresponding supervision target comprised the five joint-moment sequences over the entire window, denoted as $Y\in R^{5\times 105}$ .

Because consecutive windows overlapped, a given original time point could receive predictions from multiple windows. To reconstruct the continuous joint-moment sequence for each trial, the predictions from each window were mapped back to their corresponding temporal indices in the original trial according to the window position. Multiple predictions assigned to the same time point were combined using an equally weighted arithmetic mean to obtain a single final prediction. This reconstruction was performed independently for each trial.

After quality-control screening, six valid stair-ascent and six stair-descent trials were retained per participant. For model training, continuous synchronized data from the first valid right-foot force-plate contact onward were used without gait-cycle segmentation. All subsequent right-foot stance phases were fully captured by force plates, yielding approximately 9,000 valid time points per participant. For performance evaluation, 18 complete right-limb gait cycles were extracted per participant: six stair-ascent, six stair-descent, and six level-walking cycles from the subsequent level-walking segments of the stair-descent trials.

### 2.6 Deep learning network architecture

Predicting multi-joint and multi-planar lower-limb moments from sparse IMU data is a complex, nonlinear temporal mapping task. To support practical applicability and real-time inference, the design of the deep learning architecture addresses three primary considerations:

1. Temporal continuity and causal constraints: Human gait exhibits significant temporal continuity and long-term dependency, where the joint moment at the current moment is heavily influenced by past kinematic states. Meanwhile, to meet the real-time monitoring requirements of wearable devices, the model must strictly adhere to the principle of causality to prevent future data leakage.
2. Synergistic coupling of multiple lower limb joints: The lower limb functions as a coordinated multisegment system, in which the kinetic behaviors of the hip, knee, and ankle are temporally and mechanically coupled during locomotion. This coordination requires the model to process joint moments collectively rather than in isolation, extracting global features across joints and planes in a unified feature space to learn and leverage the implicit kinematic constraints among joints.
3. Spatiotemporal dynamic coupling of gait features: During different phases of the gait cycle, the reliance of various joint moments on specific feature channels changes dynamically. Therefore, the model requires the ability to dynamically filter feature channels along the temporal dimension, thereby enabling precise and specialized decoupling of multi-planar moment features.

Based on these considerations, we propose a Multi-task Temporal Convolutional Network, whose overall architecture is illustrated in Figure 3.

**FIGURE 3:**
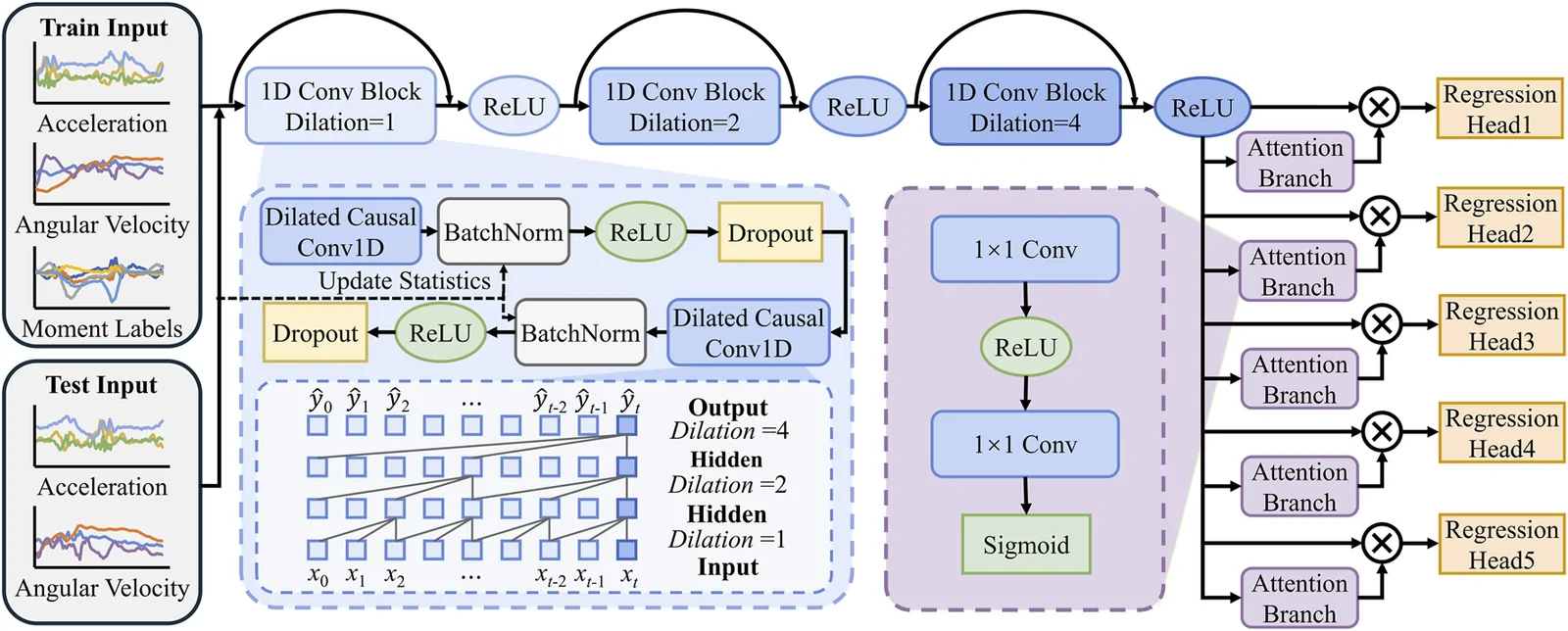
Architecture of the proposed Multi-Task Temporal Convolutional Network for continuous lower-limb joint moment prediction.

To effectively capture the long-term dependencies of the gait cycle and satisfy the real-time requirements of wearable devices, this study adopts the Temporal Convolutional Network (TCN) as the backbone. Through a causal convolution structure, the TCN prevents the leakage of future time-step data. By incorporating a dilated convolution mechanism, the TCN exponentially expands the historical receptive field of the model without significantly increasing the parameter count (Molinaro et al., 2022; Bai et al., 2018). When processing such long-sequence kinematic data, traditional recurrent neural networks like LSTM have been widely applied (Zhang et al., 2022; Wang F. et al., 2023). However, constrained by their serial computation mechanisms, they suffer from higher inference latency and are susceptible to gradient vanishing issues when handling extended sequences. Although the recently popular Transformer architecture demonstrates powerful performance, its massive computational overhead for self-attention is difficult to adapt to power-constrained wearable edge devices. In contrast, the purely convolutional architecture of the TCN supports large-scale parallel computation, offering lower memory consumption and excellent gradient stability.

To address the synergistic coupling of the lower limb multi-joint system, this study discards the conventional single-task modeling approach that constructs independent TCN feature extractors for the five moment branches. Instead, a hard parameter sharing mechanism is adopted (Wang H. et al., 2023). Lower extremity gait is fundamentally a coordinated movement driven by the same musculoskeletal system. Employing hard sharing substantially reduces the model parameter count and mitigates the risk of overfitting. More importantly, it compels the lower layers of the network to learn and extract the universal physical principles of the cross-joint kinematic chain within a unified feature space.

The TCN backbone consists of cascaded residual blocks, with the number of residual blocks, kernel size, and number of convolutional filters selected independently within each outer fold using the inner-loop validation procedure described in Section 2.9. Each residual block contains two computational layers featuring one-dimensional dilated causal convolutions and batch normalization, complemented by ReLU and Dropout layers to prevent overfitting. The network introduces an asymmetric left-padding strategy during convolution operations to ensure strict causality. For a backbone containing $N_{b}$ residual blocks, the dilation factor for the *i*th residual block, where $i\in \left\{1,\ldots ,N_{b}\right\}$ , is defined as $d_{i}=2^{i-1}$ . Accordingly, the effective receptive field depends on the architecture selected for the corresponding outer fold. The corresponding hyperparameter search spaces and selection results are summarized in Table 1. Processed by the backbone network, the 24-dimensional sparse IMU signals are mapped into a high-dimensional shared feature tensor $H\in R^{B\times C_{f}\times T}$ containing cross-joint synergistic kinetic information.

**TABLE 1:** Hyperparameter search spaces and most frequently selected values across the 14 outer folds.

Hyperparameter	Stage 1 search space	Stage 2 search space	Most frequently selected value
Number of residual blocks	2, 3, 4	—	3
Kernel size	5, 7, 9	—	7
Number of filters per layer	16, 32, 64	—	32
Learning rate	—	2.5 × 10^−4^, 5 × 10^−4^, 7.5 × 10^−4^, 1 × 10^−3^	7.5 × 10^−4^
Dropout rate	—	0.3, 0.5, 0.6	0.5

Although the TCN backbone extracts high-quality global features, the essential features required by different moment prediction tasks vary, and these requirements change dynamically with the gait phase. To address this, the study introduces an independent point-wise channel attention module (PWAttn) for each prediction task k , where k ranges from one to 5 (Dai et al., 2021).

This module preserves complete temporal resolution and infers feature importance independently at each time step through a channel-dimensional bottleneck structure composed of two layers of 1 × 1 convolutions. The dynamic weight is formulated as shown in Equation 1:

$$W^{\left(k\right)}=\sigma (W_{2}^{\left(k\right)}\delta {\left(W_{1}^{\left(k\right)}H\right))\in \left(0,1\right)}^{B\times C_{f}\times T}$$ (1)

where $W_{1}^{\left(k\right)}$ and $W_{2}^{\left(k\right)}$ represent the weight matrices for the dimensionality reduction and expansion convolution operations, δ denotes the ReLU activation function, and σ denotes the Sigmoid gating function. The calculated dynamic weight $W^{\left(k\right)}$ undergoes element-wise multiplication with the shared feature H , as expressed in Equation 2:

$$H^{\left(k\right)}=H⊙W^{\left(k\right)}$$ (2)

This mechanism enables each prediction branch to adaptively filter the most discriminative feature subspace under the current gait phase.

Upon acquiring the task-specific features, the dedicated regression head for each task operates frame-by-frame along the temporal dimension on this sequence. This process maps the high-dimensional features into the final moment prediction sequence ${\hat{y}}_{k}\in R^{B\times T}$ .

During the multi-task joint optimization phase, a fixed-weight mean squared error is adopted as the loss function. Because all joint-moment target labels were normalized before training, identical optimization weights were assigned to the five prediction tasks. The total loss L is defined as the sum of the mean squared errors across the five prediction branches, as expressed in Equation 3:

$$L=\sum_{k=1}^{5}\lambda_{k}\left(\frac{1}{T}\sum_{t=1}^{T}{\left({\hat{y}}_{k,t}-y_{k,t}\right)}^{2}\right)$$ (3)

Here, the current task weights are uniformly set to $\lambda_{k}=1$ . This configuration simplifies the hyperparameter tuning procedure while ensuring the stable convergence of the network across multi-dimensional biomechanical objectives.

### 2.7 Adaptive batch normalization for target-subject adaptation

Inter-subject differences in anthropometry and movement patterns may lead to a certain degree of feature-distribution shift between the test data and the training data, thereby degrading cross-subject prediction performance. To reduce this distribution mismatch, adaptive batch normalization (AdaBN) was introduced for unlabeled target-subject adaptation. AdaBN updates the feature statistics of batch-normalization layers using unlabeled target-domain data while keeping all trainable model parameters fixed, thereby adapting the feature statistics to the data distribution of the target participant (Li et al., 2016; 2018). Similar statistics-based adaptation methods have also been applied to biomedical time-series tasks, such as cross-dataset automatic sleep staging (Fan et al., 2022).

For each held-out target participant, an independent 10-s segment of continuous level-walking data was additionally collected during the same experimental session and reserved specifically for target-subject adaptation. The participant performed this task at a self-selected comfortable speed while wearing the four IMUs. Only IMU signals were used for adaptation, and no reference joint-moment labels were required. The adaptation segment was divided using the same windowing scheme as the model input and processed sequentially with a batch size of 32.

Before adapting to each target participant, the corresponding unadapted outer-fold model trained on the 13 development participants was reloaded. The running statistics of the batch-normalization layers in the TCN backbone were then reset and re-estimated using forward passes of the unlabeled target-subject adaptation data. During this process, all trainable model parameters remained fixed, and no gradient-based parameter updates were performed. After adaptation, the resulting target-subject-specific batch-normalization statistics were fixed and used for subsequent prediction during level walking, stair ascent, and stair descent.

Adaptation was performed independently for each outer test participant. Before adapting to the next participant, the corresponding unadapted model was reloaded to prevent information transfer across target participants. The 10-s adaptation segment was independent of the subsequent test data and was excluded from performance evaluation. For future practical deployment, this procedure could provide a simple calibration strategy in which the user performs approximately 10 s of natural level walking after donning the sensors, without requiring reference joint-moment measurements.

### 2.8 Baseline models and ablation study

#### 2.8.1 Baseline models

To evaluate the predictive performance of the proposed framework, we included an LSTM baseline and reproduced five recently published models for lower-limb joint moment estimation, including Kinetics-FM-DLR-Net (Hossain et al., 2023), XCM (Altai et al., 2023), Attention CNN-BiLSTM (Liang et al., 2023), TCN-BiLSTM (Wang F. et al., 2023), and TCN-xLSTM (Tang et al., 2025). Among these models, several employ hybrid convolutional–recurrent architectures, which have also been explored in related wearable biomechanical estimation tasks, such as CNN-BiLSTM-based dynamic plantar-pressure estimation from inertial sensors (Qian et al., 2025).

For the published models, the main network architectures were retained from the corresponding original studies without architecture-level hyperparameter search, with only the input and output layers adapted to the present prediction task. For the external baseline comparison, all external baseline models and MTCN-Attn used the same IMU inputs and prediction targets and were evaluated using the same data-processing and cross-validation procedures. MTCN-Attn without target-subject adaptation was used for statistical comparisons with the six external baseline models, whereas the incremental effect of AdaBN was evaluated separately through comparison between MTCN-Attn and AdaBN-MTCN-Attn in the ablation analysis.

The main network architectures and hyperparameter settings of the baseline models are summarized in Table 2.

**TABLE 2:** Architecture and hyperparameter settings of the baseline models.

Model	Architecture and hyperparameter settings
LSTM	Two-layer unidirectional LSTM; hidden size = 128; dropout = 0.6
Kinetics-FM-DLR-Net	Input batch normalization; auxiliary supervision; deep linear residual (DLR) module; dropout = 0.3
XCM	Number of filters = 128; window percentage = 1.0
Attention CNN-BiLSTM	Conv1D with 128 filters and kernel size = 1; BiLSTM hidden size = 64; dropout = 0.5
TCN-BiLSTM	TCN channels = 8; kernel size = 1; dilation factors = [1, 2]; BiLSTM hidden size = 8; dropout = 0.5
TCN-xLSTM	TCN channels = [256, 128, 64]; kernel size = 20; dilation factors = [1, 2, 4]; embedding dimension = 24; attention heads = 4; dropout = 0.3

#### 2.8.2 Ablation study

To evaluate the contribution of each component of the proposed framework, four model variants were constructed by progressively introducing multi-task hard parameter sharing, point-wise channel attention, and AdaBN: ST-TCN, MT-TCN, MTCN-Attn, and AdaBN-MTCN-Attn.

ST-TCN consisted of five independent TCN models, each predicting one joint moment, and served as the reference model for the ablation study. MT-TCN replaced the independent TCNs with a hard-shared TCN backbone and task-specific prediction heads to evaluate the effect of multi-task hard parameter sharing. MTCN-Attn further incorporated a point-wise channel attention module into each prediction branch to evaluate its contribution to multi-joint and multi-planar joint moment prediction. Finally, AdaBN-MTCN-Attn further introduced AdaBN during testing to evaluate the contribution of target-subject adaptation to cross-subject prediction performance.

For ST-TCN, MT-TCN, and MTCN-Attn, hyperparameters were selected using the same inner-loop validation procedure within each outer fold, as described in Section 2.9. All model variants used the same data partitions and evaluation procedures, allowing the incremental contribution of each added component to be evaluated through comparisons between consecutive models.

### 2.9 Model training and nested cross-validation

All models were implemented in PyTorch and trained on a single NVIDIA GeForce RTX 5080 GPU using the Adam optimizer. Random seeds were fixed throughout the experiments to improve reproducibility.

Cross-subject generalization was evaluated using nested subject-level cross-validation. The outer loop consisted of 14-fold leave-one-subject-out (LOSO) cross-validation, with one participant held out as the independent test subject and the remaining 13 participants constituting the development set. All model-selection procedures were performed exclusively within the development set of each outer fold.

Within each outer fold, the 13 development participants were divided into three mutually exclusive and approximately balanced subject-level inner folds. Each inner fold served once as the validation set, while the remaining folds were used for training. Hyperparameter optimization was performed independently within each outer fold using only the corresponding inner-training and validation data.

To limit the computational cost of hyperparameter optimization, a two-stage hyperparameter search strategy was adopted, similar to the staged TCN optimization used in previous joint-moment estimation research (Molinaro et al., 2024b). In the first stage, architectural hyperparameters, including the number of residual blocks, kernel size, and number of convolutional filters, were optimized. In the second stage, the selected architecture was fixed while the learning rate and dropout rate were further optimized. The candidate ranges are summarized in Table 1. Each candidate configuration was trained for 100 epochs, and the mean validation RMSE across the three inner folds at each epoch was used to determine the optimal hyperparameters and training duration for the corresponding outer fold.

After inner-loop optimization, the final model was trained on all 13 development participants using the selected hyperparameters and training duration and was subsequently evaluated on the held-out outer test subject. This procedure was repeated independently across all 14 outer folds so that each participant served once as the independent test subject.

To prevent data leakage, Z-score normalization parameters for the IMU inputs and joint-moment labels were estimated from the corresponding training data at each stage and subsequently applied to the validation or test data.

For the external baseline comparison, all models were evaluated under the same nested cross-validation framework. The Adam optimizer, a maximum of 100 training epochs, and the same learning-rate search range (2.5 × 10^-4^–1 × 10^-3^) were used for all models. For each outer fold, the epoch with the lowest mean inner-validation RMSE was selected as the final training duration, while model-specific architectural parameters followed the corresponding original studies.

In addition, supplementary evaluation was performed using the open-source lower-limb biomechanics dataset reported by Camargo et al. (2021). Data from all 22 participants were included, comprising level walking, stair ascent, and stair descent. Four IMUs were used as model inputs, and four joint moments were evaluated: hip flexion/extension, hip adduction/abduction, knee flexion/extension, and ankle dorsiflexion/plantarflexion. The same model framework was applied, with only the input and output interfaces adapted to the public dataset. Model training, hyperparameter selection, and testing were performed independently within the Camargo dataset using the same nested subject-level cross-validation procedure described above.

### 2.10 Model evaluation

We used the root mean square error (RMSE) and the coefficient of determination (R^2^) as quantitative evaluation metrics. RMSE quantifies the absolute deviation between the predicted and reference joint moments, whereas R^2^ reflects the model’s ability to explain the variation in the reference joint moments. All metrics were calculated from the reconstructed, non-duplicated prediction sequences, with the participant treated as the independent statistical unit.

Model complexity was evaluated using the number of trainable parameters and multiply–accumulate operations (MACs) per input window. Inference latency was measured using single-window forward passes with a batch size of one on an NVIDIA GeForce RTX 5080 GPU, and the mean ± standard deviation over 500 repeated measurements was reported.

Statistical comparisons between models were based on participant-level overall R^2^. All paired model comparisons were performed using two-sided exact paired permutation tests. The mean paired difference was reported as the effect estimate, together with its bias-corrected and accelerated (BCa) bootstrap 95% confidence interval. Holm correction was applied separately to the six comparisons between MTCN-Attn and the external baseline models and to the three sequential ablation comparisons. Statistical significance was set at p < 0.05, and Holm-adjusted p values were reported.

## 3 Results

Figures 4, 5 illustrate the joint-moment prediction performance of the four model configurations in terms of RMSE and $R^{2}$ , respectively. The proposed AdaBN-MTCN-Attn framework demonstrated stable prediction performance across the three locomotion modes. In terms of overall task performance, the model achieved the highest $R^{2}$ during level walking ( $R^{2}=0.781\pm 0.078$ ), followed by stair descent ( $R^{2}=0.764\pm 0.063$ ) and stair ascent ( $R^{2}=0.729\pm 0.061$ ). Across all three locomotion modes and five prediction targets, AdaBN-MTCN-Attn achieved an overall $R^{2}$ of 0.759±0.032 and an overall RMSE of 0.136±0.014 N·m/kg.

**FIGURE 4:**
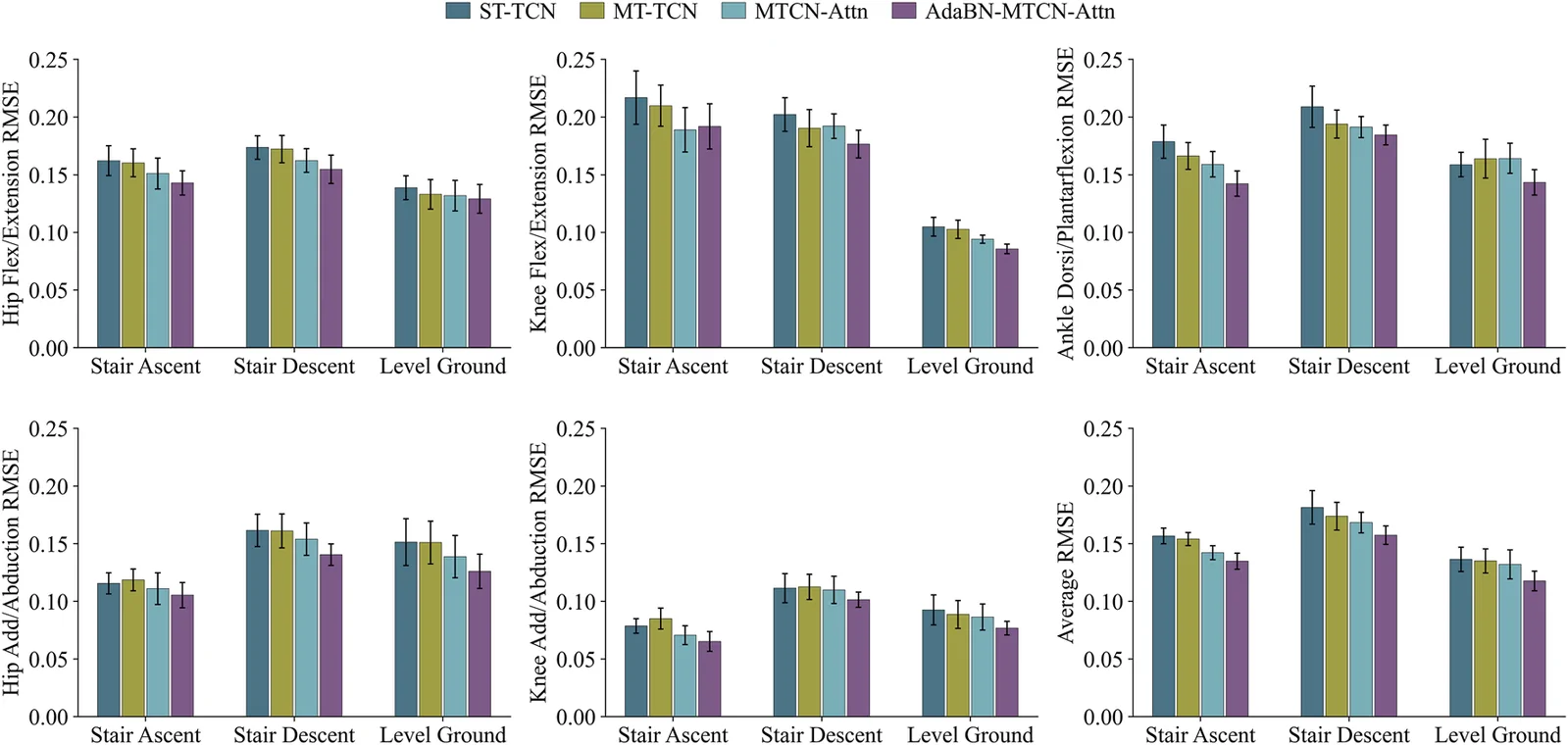
Comparison of prediction errors (RMSE) among four models for five joint moments across three locomotion modes. Bar heights and error bars represent the mean and standard deviation across subjects. The bottom-right subplot shows the overall average RMSE. All values are in N·m/kg.

**FIGURE 5:**
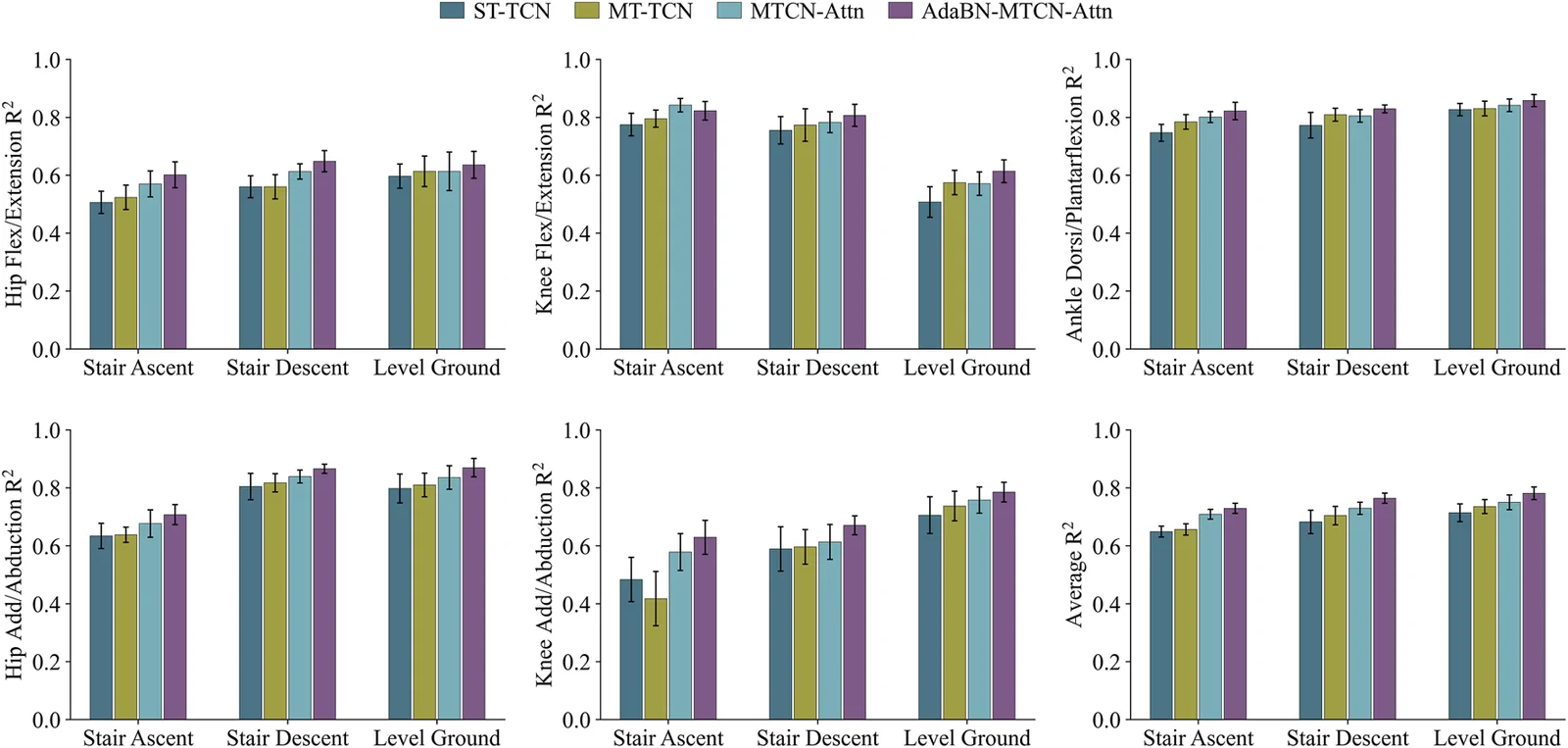
Comparison of coefficients of determination (R^2^) among four models for five joint moments across three locomotion modes. Bar heights and error bars represent the mean and standard deviation across subjects. The bottom-right subplot shows the overall average R^2^.

For sagittal-plane moment prediction, the ankle dorsiflexion/plantarflexion moment showed the highest prediction performance ( $R^{2}=0.822$ – 0.858 ), whereas the hip flexion/extension moment showed relatively lower performance ( $R^{2}=0.601$ – 0.648 ). For coronal-plane moment prediction, the hip adduction/abduction moment ( $R^{2}=0.707$ – 0.870 ) was generally predicted more accurately than the knee adduction/abduction moment ( $R^{2}=0.629$ – 0.785 ). Prediction performance also varied across locomotion modes. Specifically, the $R^{2}$ values for the knee flexion/extension moment exceeded 0.80 during both stair ascent and stair descent, whereas performance was lower during level walking ( $R^{2}=0.613$ ). Conversely, the hip adduction/abduction moment was predicted more accurately during level walking and stair descent than during stair ascent. Overall, the proposed framework achieved an $R^{2}$ above 0.60 for all 15 joint-task combinations.

To further evaluate predictive performance relative to existing methods, Table 3 compares MTCN-Attn with six external baseline models. Among the models evaluated without target-subject adaptation, MTCN-Attn achieved the highest numerical overall R^2^ (0.727 ± 0.049) and significantly outperformed LSTM, XCM, TCN-BiLSTM, and TCN-xLSTM after Holm correction, whereas the differences relative to Kinetics-FM-DLR-Net and Attention CNN-BiLSTM were not statistically significant. With target-subject adaptation, AdaBN-MTCN-Attn further increased the overall R^2^ to 0.759 ± 0.032. Both proposed variants contained 53.4 K trainable parameters and required 5.50 M MACs per input window, with a measured single-window inference latency of 1.672 ± 0.146 ms.

**TABLE 3:** Overall prediction performance and computational characteristics of the proposed models and external baselines.

Model	Overall RMSE (N·m/kg)	Overall R^2^	ΔR^2^ [95% CI]	Holm-adjusted p	Parameters	MACs/window	Latency (ms)
LSTM	0.160 ± 0.023	0.674 ± 0.052	0.052 [0.032, 0.082]	0.003	345.7 K	22.07 M	1.289 ± 0.190
Kinetics-FM-DLR-Net	0.152 ± 0.030	0.714 ± 0.071	0.013 [-0.012, 0.033]	0.573	20.73 M	751.60 M	12.310 ± 0.894
XCM	0.164 ± 0.022	0.681 ± 0.041	0.046 [0.020, 0.067]	0.014	686.3 K	103.37 M	0.445 ± 0.059
Attention CNN-BiLSTM	0.152 ± 0.028	0.715 ± 0.053	0.012 [-0.013, 0.032]	0.573	1.46 M	13.40 M	0.386 ± 0.080
TCN-BiLSTM	0.169 ± 0.024	0.666 ± 0.047	0.061 [0.032, 0.091]	0.007	169.9 K	0.336 M	0.685 ± 0.115
TCN-xLSTM	0.160 ± 0.023	0.692 ± 0.040	0.035 [0.019, 0.054]	0.007	4.08 M	291.33 M	43.552 ± 1.382
MTCN-Attn (Ours)	0.148 ± 0.025	0.727 ± 0.049	Reference	—	53.4 K	5.50 M	1.672 ± 0.146
AdaBN-MTCN-Attn (Ours)	0.136 ± 0.014	0.759 ± 0.032	—	—	53.4 K	5.50 M	1.672 ± 0.146

Values are participant-level mean ± SD (n = 14). ΔR^2^ = MTCN-Attn − baseline and is reported as the mean paired difference with a BCa bootstrap 95% CI. Holm correction was applied across the six external baseline comparisons. The effect of AdaBN, relative to MTCN-Attn is reported in Table 4. For MTCN-Attn and AdaBN-MTCN-Attn, the reported complexity metrics correspond to the most frequently selected architecture across the 14 outer folds: three residual blocks, kernel size 7, and 32 filters per layer.

To further quantify the contribution of each network component, Table 4 presents the sequential ablation results obtained by progressively introducing multi-task hard parameter sharing, point-wise channel attention, and AdaBN. Introducing hard parameter sharing increased the overall performance from ST-TCN ( $R^{2}=0.684\pm 0.050$ ) to MT-TCN ( $R^{2}=0.701\pm 0.041$ ), corresponding to a paired difference of $\Delta R^{2}=0.017$ [95% CI: 0.006, 0.028] (Holm-adjusted p=0.021 ). Further incorporating point-wise channel attention increased the overall $R^{2}$ to 0.727±0.049 , with a further paired difference of $\Delta R^{2}=0.026$ [0.011, 0.040] relative to MT-TCN (Holm-adjusted p=0.021 ). Finally, target-subject adaptation using AdaBN at test time increased the overall $R^{2}$ to 0.759±0.032 , corresponding to a paired difference of $\Delta R^{2}=0.032$ [0.015, 0.058] relative to MTCN-Attn (Holm-adjusted p=0.021 ). Thus, each sequentially introduced component produced a statistically significant improvement in prediction performance.

**TABLE 4:** Overall performance and sequential ablation analysis of the proposed framework.

Model	Overall RMSE (N·m/kg)	Overall R^2^	ΔR^2^ vs. preceding model [95% CI]	Holm-adjusted p
ST-TCN	0.157 ± 0.020	0.684 ± 0.050	Reference	—
MT-TCN	0.153 ± 0.020	0.701 ± 0.041	0.017 [0.006, 0.028]	0.021
MTCN-Attn	0.148 ± 0.025	0.727 ± 0.049	0.026 [0.011, 0.040]	0.021
AdaBN-MTCN-Attn	0.136 ± 0.014	0.759 ± 0.032	0.032 [0.015, 0.058]	0.021

Values are participant-level mean ± SD (n = 14). ΔR^2^ represents the mean paired difference between each model and the immediately preceding model, with BCa bootstrap 95% CIs. Holm correction was applied across the three sequential ablation comparisons.

In addition, to evaluate the applicability of the proposed framework to an independent data source, supplementary evaluation was performed using the public lower-limb biomechanics dataset reported by Camargo et al. (2021). As shown in Table 5, the model maintained favorable prediction performance across the four available joint-moment targets ( $R^{2}=0.704$ – 0.840 ). The ankle dorsiflexion/plantarflexion and knee flexion/extension moments showed relatively higher prediction performance ( $R^{2}=0.840\pm 0.062$ and 0.824±0.049 , respectively). These results further support the applicability of the proposed framework on an independently collected public dataset.

**TABLE 5:** Joint-moment prediction performance on the Camargo et al. (2021) public dataset.

Joint moment	RMSE (N·m/kg)	R^2^
Hip flexion/extension	0.196 ± 0.037	0.794 ± 0.056
Hip adduction/abduction	0.162 ± 0.056	0.704 ± 0.136
Knee flexion/extension	0.214 ± 0.037	0.824 ± 0.049
Ankle dorsiflexion/plantarflexion	0.206 ± 0.039	0.840 ± 0.062

Values are mean ± SD, across participants (n = 22).

Figure 6 shows the reference and predicted joint moments for a representative participant during level walking, stair ascent, and stair descent.

**FIGURE 6:**
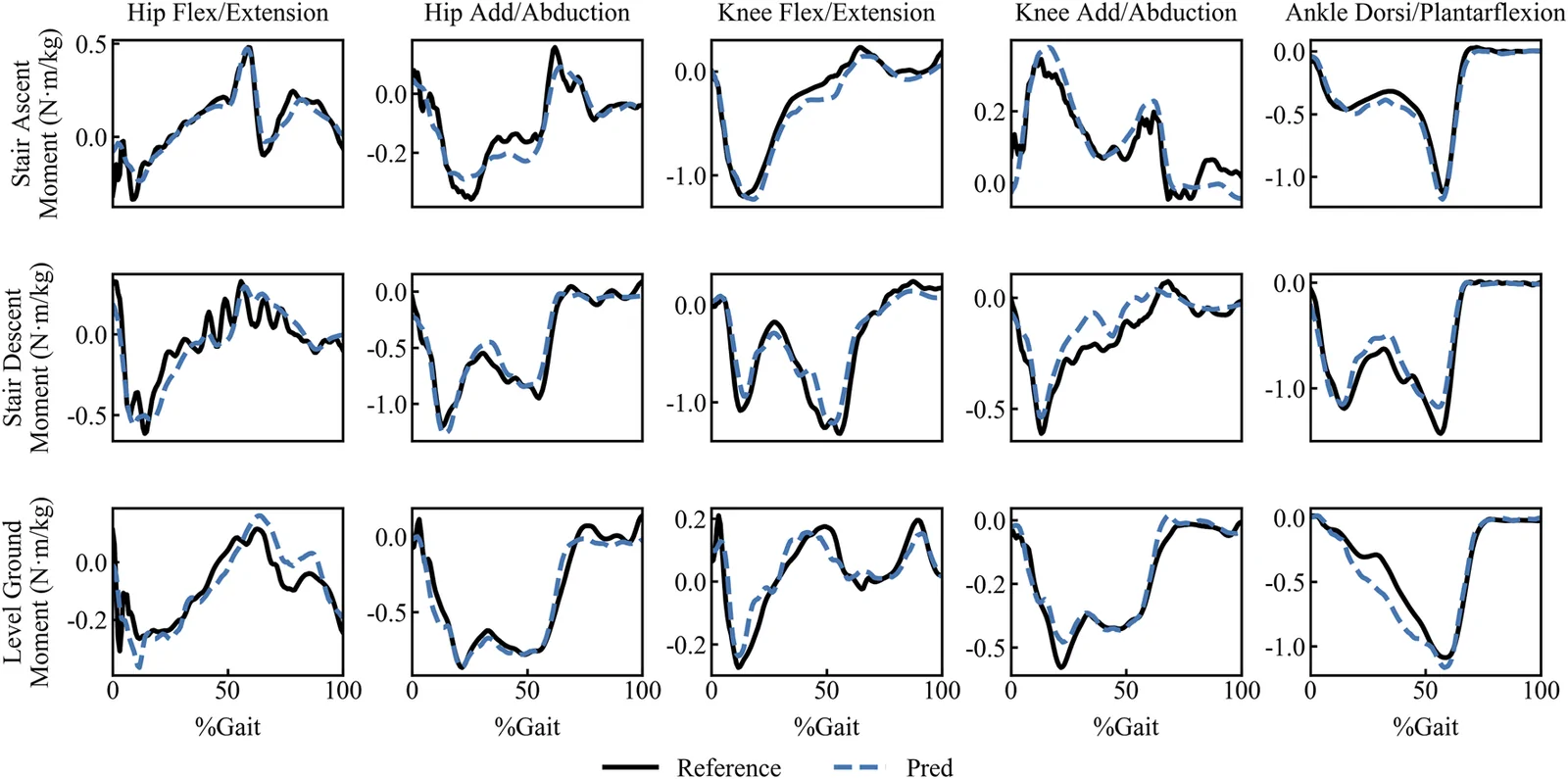
Time-series tracking of joint moments across different tasks and joints. The curves compare AdaBN-MTCN-Attn predictions against the reference over a 0%–100% normalized gait cycle.

## 4 Discussion

This study developed a wearable framework based on a sparse inertial measurement unit configuration for estimating sagittal- and coronal-plane joint moments of the hip, knee, and ankle. By integrating a temporal convolutional network with a hard-parameter-sharing backbone, a point-wise channel attention mechanism, and test-stage adaptive batch normalization, the proposed framework achieved continuous prediction of five joint moments during level walking, stair ascent, and stair descent. The proposed multi-task architecture showed competitive predictive performance relative to the external baseline models, and target-subject adaptation further improved the overall prediction accuracy without requiring reference joint-moment labels.

The proposed multi-task architecture, MT-TCN, demonstrated favorable parameter efficiency. Under the backbone configuration with three residual blocks, a kernel size of 7, and 32 filters per layer, the five independent ST-TCN models contained 218.0 K trainable parameters in total, compared with 48.0 K for MT-TCN. Hard parameter sharing also increased the overall $R^{2}$ from 0.684±0.050 to 0.701±0.041 , corresponding to a paired difference of $\Delta R^{2}=0.017$ [95% CI: 0.006, 0.028], which remained statistically significant after Holm correction ( p=0.021 ). This improvement is consistent with the biomechanical understanding that hip, knee, and ankle moments are coordinated outputs of an interconnected multisegment system. The shared backbone can therefore extract common temporal patterns across joints and anatomical planes within a unified feature space.

The introduction of point-wise channel attention increased the overall $R^{2}$ from 0.701±0.041 to 0.727±0.049 , corresponding to a paired difference of $\Delta R^{2}=0.026$ [0.011, 0.040] (Holm-adjusted p=0.021 ). This result indicates that time-varying channel reweighting for different prediction tasks helps each prediction branch select information from the shared representation that is more relevant to the corresponding joint moment. The inertial features relevant to different joints and anatomical planes are not identical. In particular, sagittal- and coronal-plane movements differ in their motion amplitudes and kinetic characteristics, and different prediction tasks may therefore rely on different subsets of the shared features. Point-wise channel attention enables task-specific feature selection while retaining the shared representation, thereby improving joint prediction of multi-joint and multi-planar moments.

Test-stage adaptive batch normalization further increased the overall $R^{2}$ from 0.727±0.049 to 0.759±0.032 , corresponding to a paired difference of $\Delta R^{2}=0.032$ [0.015, 0.058] (Holm-adjusted p=0.021 ). This result suggests that recalibrating the feature statistics of the batch-normalization layers can reduce part of the cross-subject distribution mismatch. In this study, an additional 10-s segment of unlabeled level-walking IMU data from each target participant was used to reset and re-estimate the running statistics of the batch-normalization layers, while all trainable model parameters remained fixed. Thus, target-subject adaptation required neither reference joint-moment labels nor gradient-based parameter updates. Inter-subject differences in anthropometric characteristics and movement patterns, together with variations in sensor attachment under practical use, may alter the statistical distributions of the inertial signals and intermediate features. Statistics-based adaptation allows these feature statistics to be recalibrated for the target participant. The extent to which such adaptation can compensate for sensor-placement variability remains to be evaluated.

Although the absolute performance gains from the individual components were modest, these improvements did not increase the sensing burden and introduced only limited additional model complexity, which may still be relevant for wearable deployment. However, the clinical relevance of these small reductions in prediction error remains to be established in application-specific evaluations.

Under the present evaluation framework, MTCN-Attn showed competitive predictive performance relative to the external baseline models. MTCN-Attn achieved the highest numerical overall R^2^ among the models evaluated without target-subject adaptation and significantly outperformed four of the six external baselines. Several substantially larger baseline models did not achieve higher predictive performance under the present comparison setting. These findings suggest that, for the present task, a compact architecture tailored to multi-joint prediction can remain competitive with substantially larger models. Moreover, although the inference latency of MTCN-Attn was not the lowest among all baseline models, its single-window inference time remained in the millisecond range and was well below the inter-sample interval at a 100-Hz sampling rate, supporting the feasibility of real-time inference under the current hardware configuration.

Beyond the model architecture, sensor configuration represents another important consideration for practical wearable implementation. Sensor-configuration ablation (Table 6) showed that the complete four-IMU configuration achieved the highest overall prediction performance and that removing any sensor reduced performance for at least some outputs. The thigh and shank sensors were particularly important for knee-moment estimation. Removing the shank IMU caused the largest reduction in knee adduction moment performance, with $R^{2}$ decreasing from 0.697 to 0.542, whereas removing the thigh IMU caused the largest numerical reduction in knee flexion/extension performance, from 0.779 to 0.638. These findings suggest that the thigh and shank sensors provide complementary information for knee-moment prediction. Removing the thigh IMU also produced broad reductions across hip, knee, and ankle outputs, while the pelvis IMU contributed particularly to hip flexion/extension prediction. The thigh-shank configuration produced results numerically close to the four-sensor configuration for several outputs other than hip flexion/extension. One possible explanation for the limited benefit of adding the foot sensor in some conditions is the presence of impact-related components in foot-mounted inertial signals; however, this hypothesis was not directly tested. Overall, the results suggest that a thigh-shank configuration may provide a favorable balance between sensor burden and prediction performance when hip flexion/extension is not the primary outcome. This potential trade-off should be evaluated prospectively using predefined accuracy and usability criteria.

**TABLE 6:** Sensor configuration ablation in terms of R^2^.

Sensor configuration	Hip FE	Hip AA	Knee FE	Knee AA	Ankle DP
IMU 1 + 2 + 3 + 4	0.624 ± 0.14	0.819 ± 0.12	0.779 ± 0.13	0.697 ± 0.14	0.837 ± 0.07
IMU 1 + 2 + 3	0.557 ± 0.21	0.805 ± 0.13	0.740 ± 0.21	0.622 ± 0.30	0.836 ± 0.08
IMU 1 + 2 + 4	0.571 ± 0.16	0.800 ± 0.14	0.746 ± 0.21	0.651 ± 0.20	0.824 ± 0.09
IMU 1 + 3 + 4	0.521 ± 0.19	0.751 ± 0.16	0.675 ± 0.28	0.542 ± 0.28	0.782 ± 0.12
IMU 2 + 3 + 4	0.543 ± 0.19	0.777 ± 0.17	0.638 ± 0.50	0.617 ± 0.28	0.801 ± 0.11
IMU 1 + 2	0.506 ± 0.17	0.785 ± 0.15	0.747 ± 0.19	0.648 ± 0.24	0.825 ± 0.10
IMU 1	0.433 ± 0.27	0.731 ± 0.15	0.660 ± 0.29	0.608 ± 0.18	0.745 ± 0.12
IMU 2	0.442 ± 0.24	0.760 ± 0.18	0.663 ± 0.41	0.567 ± 0.36	0.791 ± 0.08
IMU 3	0.365 ± 0.25	0.686 ± 0.18	0.432 ± 0.71	0.514 ± 0.33	0.716 ± 0.17
IMU 4	0.337 ± 0.36	0.710 ± 0.16	0.577 ± 0.52	0.420 ± 0.41	0.712 ± 0.14

IMU, locations: 1 = thigh, 2 = shank, 3 = foot, and 4 = pelvis. FE, flexion/extension; AA, adduction/abduction; DP, dorsiflexion/plantarflexion.

To further assess the applicability of the framework beyond the present dataset, supplementary validation was performed using the public lower-limb biomechanics dataset reported by Camargo et al. (2021). The model achieved $R^{2}$ values ranging from 0.704 to 0.840 across the four available joint-moment targets, with $R^{2}$ values above 0.80 for knee flexion/extension and ankle dorsiflexion/plantarflexion moments. Despite differences between the public dataset and the present dataset in data source, sensor configuration, and input/output composition, the framework maintained favorable multi-joint moment prediction performance, providing supplementary evidence for its applicability on an independently collected dataset.

Because the primary R^2^ and RMSE metrics were calculated over complete gait cycles, they mainly characterize overall waveform prediction and may not fully reflect errors in clinically relevant stance-phase features. To provide clinical context for the prediction error, the second knee adduction/abduction moment peak during level walking was additionally evaluated, yielding an RMSE of 0.079 N·m/kg. Yamagata et al. (2021) reported a difference of approximately 0.121 N·m/kg in the second knee adduction moment peak between patients with mild and severe knee osteoarthritis. The present peak-estimation error is therefore substantial relative to this previously reported between-group difference. Further validation in clinically and demographically diverse populations is needed to determine whether the estimated joint moments can reliably preserve clinically relevant between-group differences or longitudinal changes. Previous studies have evaluated IMU-derived biomechanical variables by examining whether they can capture age- or osteoarthritis-related gait differences (Hafer et al., 2022; Yu et al., 2023), providing a relevant direction for future validation of the present framework.

Several limitations of this study should be acknowledged. First, the reference joint moments were derived from laboratory-based motion capture, force-plate measurements, and OpenSim inverse dynamics, and therefore represent model-based estimates of net joint moments rather than direct *in vivo* measurements of internal joint loading. Their accuracy may be affected by experimental measurement errors and modeling assumptions. Accordingly, the moment predictions in this study should be interpreted as a portable approximation of laboratory-based inverse-dynamics estimates, with the potential to facilitate future biomechanical assessment beyond laboratory settings. Second, the current dataset comprises a limited set of laboratory locomotion tasks collected from a relatively small sample of 14 healthy young adults. Moreover, all data were collected in a single laboratory using a fixed staircase configuration, which may limit generalization to other environments and stair geometries. A further protocol-specific limitation is that the analyzed level-walking cycles were extracted approximately 0.8 m beyond the final stair, and residual effects of the preceding stair descent cannot be completely excluded. Because older adults or individuals with musculoskeletal pathologies often exhibit abnormal compensatory gaits, the current findings may not immediately generalize to these populations. Recent work has also shown that IMU-based biomechanical estimation can exhibit reduced cross-cohort generalization between healthy adults and adults with osteoarthritis (Ullah et al., 2026). Future work will necessitate the inclusion of larger and more diverse cohorts, alongside expanded testing protocols, to further train and fine-tune the model for robust clinical generalization. Additionally, constrained by the dataset size, intra-subject training and testing were not explored; however, for specific clinical applications, personalized intra-subject fine-tuning could be seamlessly integrated to further boost prediction accuracy (Tang et al., 2025).

Third, although the individual model components improved overall prediction performance, the absolute magnitude of these gains was modest, and their practical relevance requires further application-specific validation. The proposed model underwent architecture-level hyperparameter optimization, whereas the external baseline models retained their originally published architectures and received more limited tuning. This difference in tuning scope may have influenced the observed between-model performance differences. Future studies could further compare different model architectures under a unified hyperparameter-search budget. In addition, each model configuration was trained once using a fixed random seed, and variability due to random initialization was not quantified. Future work should evaluate the stability of the observed between-model differences across repeated runs with multiple random seeds. Fourth, although the second knee adduction/abduction moment peak during level walking was additionally evaluated, peak-based accuracy was not systematically assessed across other joints and locomotion modes, and joint-moment impulses were not evaluated. Future studies should further examine these feature-level measures where they are relevant to the intended biomechanical or clinical application.

Fifth, regarding feature input dimensions, the current framework does not explicitly incorporate specific anthropometric parameters, such as the participant’s height and body mass. Fusing these readily accessible anthropometric covariates with the deep learning features holds the potential to mitigate residual errors induced by inter-subject variability, which remains a clear target for our future development. Finally, the present study did not directly evaluate robustness to repeated sensor donning or controlled sensor-placement perturbations. In real-world use, variations in sensor position, orientation, and strap tightness may alter the IMU signal distributions and affect prediction performance. Future studies should therefore systematically evaluate repeated-donning and sensor-displacement conditions and investigate calibration strategies to improve robustness to these perturbations. Software-level features, such as sensor-position anomaly detection and installation guidance, may also facilitate more consistent sensor placement. These practical engineering issues remain to be addressed before out-of-laboratory or daily-living deployment.

## 5 Conclusion

This study developed a sparse wearable framework for continuous prediction of five lower-limb joint moments in the sagittal and coronal planes. The proposed architecture combined a shared temporal convolutional backbone, task-specific point-wise channel attention, and adaptive batch normalization using unlabeled target-subject data. Under nested subject-level leave-one-subject-out cross-validation, MTCN-Attn achieved the highest numerical overall R^2^ among the models evaluated without target-subject adaptation, while AdaBN further improved the prediction performance. Supplementary evaluation on an independent public dataset further supported its applicability on a separately collected dataset. Sensor-configuration analysis further indicated that the four sensor locations provided complementary information for the prediction tasks. These findings demonstrate the feasibility of multi-joint and multi-planar moment estimation using a limited number of wearable inertial sensors. Further validation in larger and more diverse populations, repeated sensor-placement conditions, clinically relevant gait patterns, and out-of-laboratory environments is required before clinical or daily-living deployment.

## Data Availability

The raw data supporting the conclusions of this article will be made available by the authors, without undue reservation.
